# Supplementary material for: Metabolomic Profiling and Characterization of a Novel 3D Culture System for Studying Chondrocyte Mechanotransduction
Source: Cell Mol Bioeng. 2025 Oct 11;18(6):589–609. doi: 10.1007/s12195-025-00872-z (PMC12664864; doi:10.1007/s12195-025-00872-z)
Supplement: Supplementary file 3 — Supplementary file3 (DOCX 3468 KB) [file 12195_2025_872_MOESM3_ESM.docx]

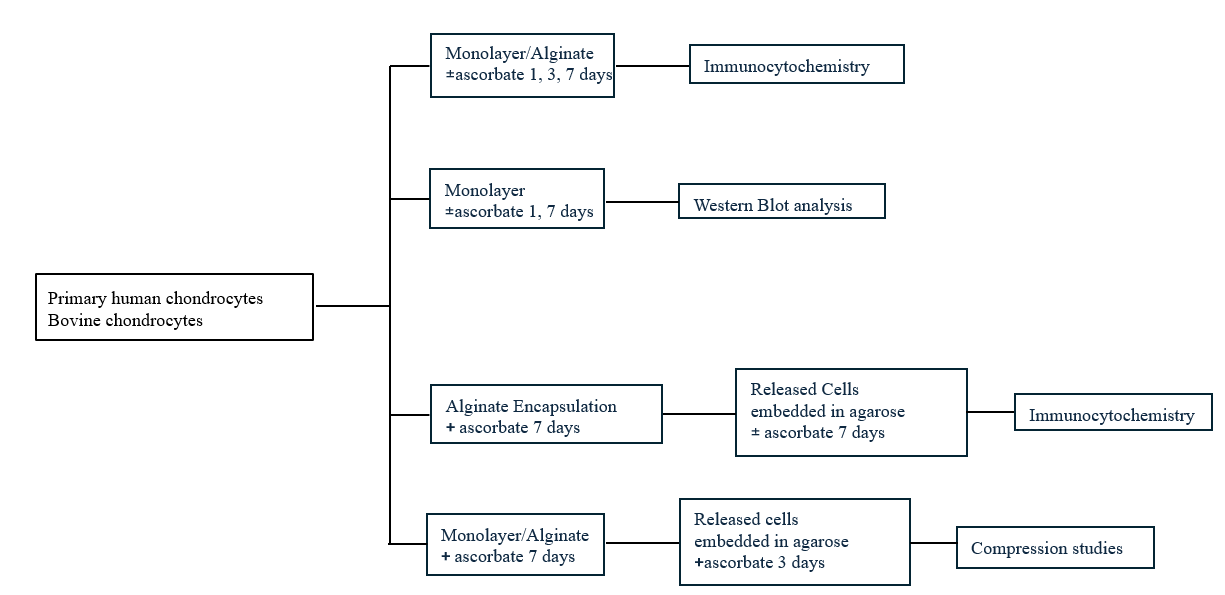


# Figure S1

**Schematic of experimental setup.** Primary human chondrocytes and bovine chondrocytes were cultured under multiple conditions to assess matrix production. Cells were maintained in monolayer or encapsulated in alginate, with or without supplementation of sodium L-ascorbate, for 1, 3, or 7 days. Monolayer and alginate cultures were analyzed by immunocytochemistry to evaluate collagen VI localization. Monolayer was cultured, with or without supplementation of sodium L-ascorbate, for 1 or 7 days for Western blot analysis to examine collagen production. A set of alginate encapsulated cells was cultured in sodium L-ascorbate for 7 days. Cells released from alginate were re-embedded in agarose and further cultured with or without ascorbate for 7 days and these constructs were subjected to immunocytochemistry. A subset of ascorbate treated monolayer and alginate encapsulated cells were then embedded in agarose and these constructs were cultured in media containing sodium L-ascorbate for 3 days before being subjected to mechanical compression testing. Concentration of sodium L-ascorbate is 50 µg/ml.

#
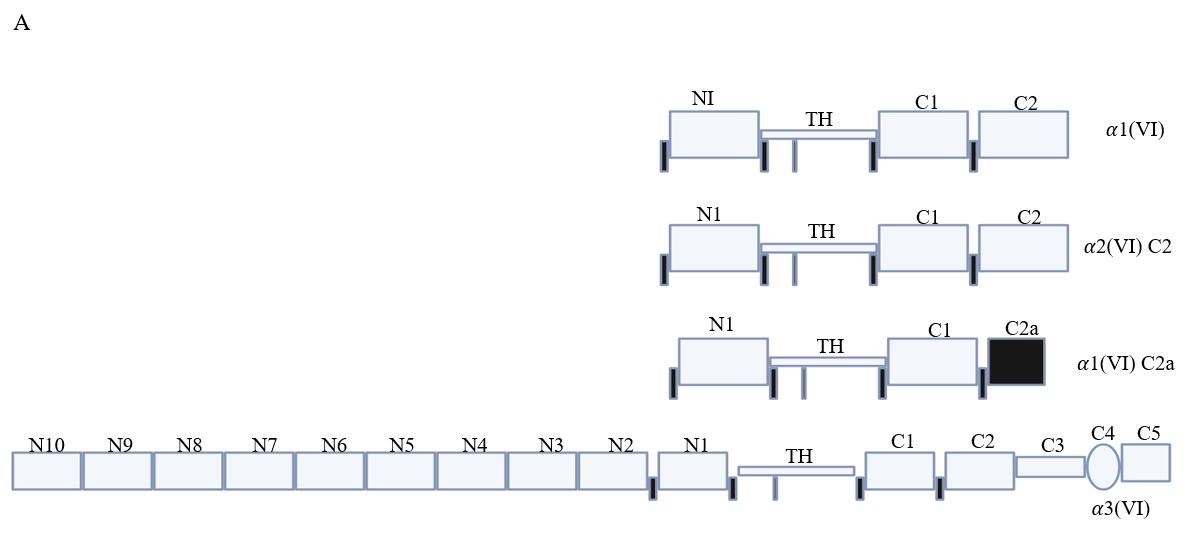

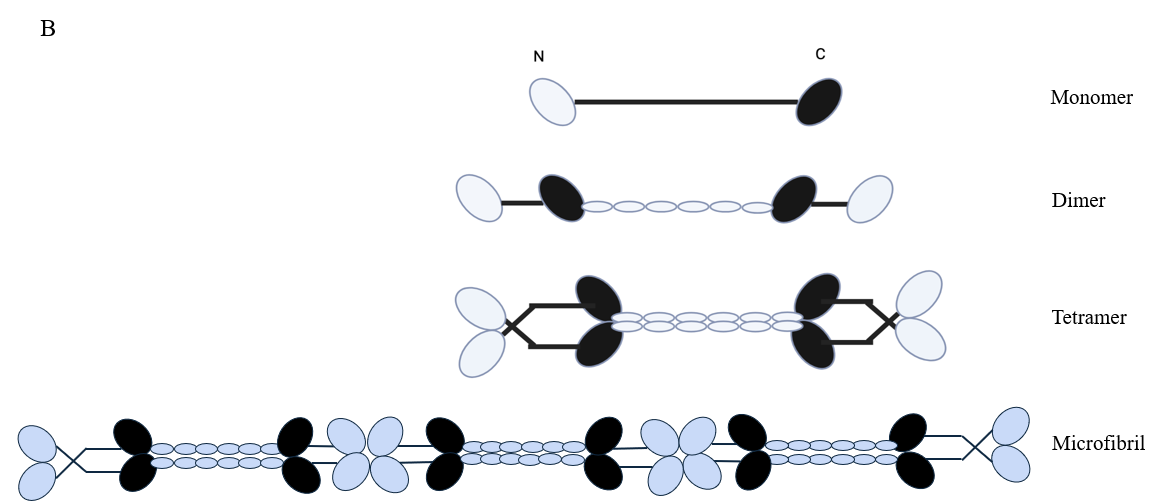


# Figure S2

**Molecular structure of Collagen VI. (A)** Shown are the three chains α1(~140kDa), α2 (~140kDa) and α3 (~300kDa) of Collagen VI. Each chain contains a triple helical domain (TH) flanked by N-and C globular domains (N1-N10). N1, N2, N4, N5, N6, C1 and C2 correspond to Von Willebrand Factor A (vWFA) domain. N3, N7, N8, N9, N10 are alternatively spliced vWFA domains. C3 is the lysine proline repeats. C4 is the Fibronectin type III motif. TH is the triple helix (Gly-X-Y). C5 is the Kunitz protease inhibitor motif. The minor chain variant 2(VI) C2a, has a shorter C2a domain at the C-terminal end compared with the major 2(VI) C2 chain, due to alternative splicing of the 3-end of the gene. **(B)** Collagen VI assembly. The three subunits- α1, α2 and α3 associate to form a triple helical monomer, followed by assembly into disulphide bonded antiparallel dimers which then align to form tetramers and are linked end-to-end into microfibrils. Adapted from Deconinck et al 2009 and Zhang et al 2010 and made in bioRender.com


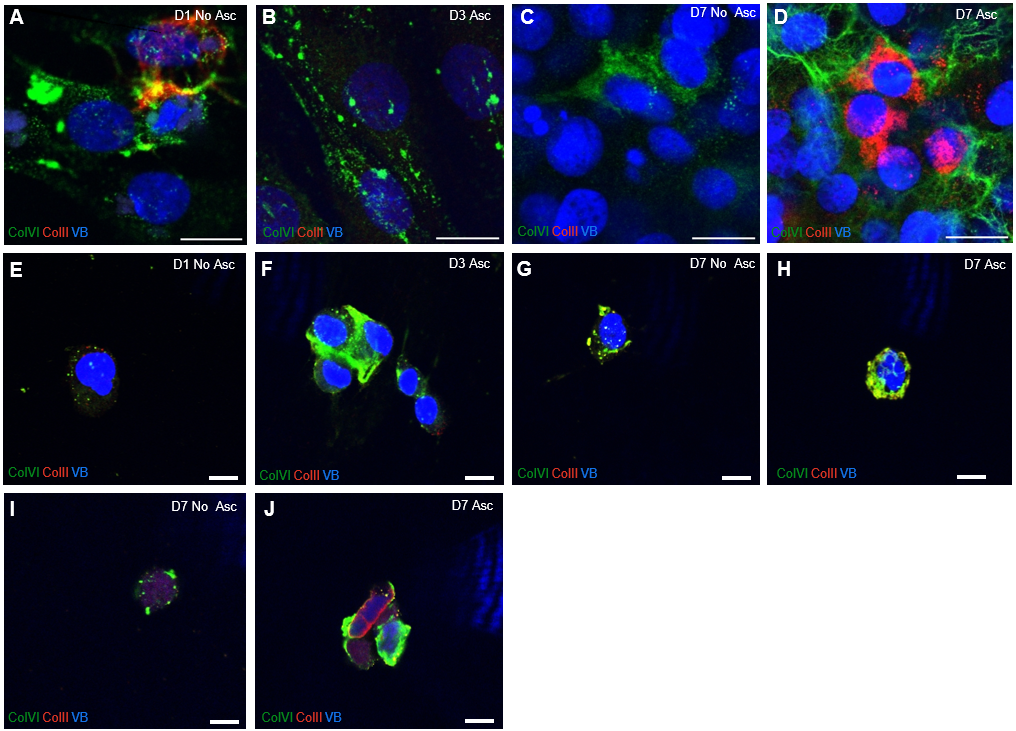


# Figure S3

**Collagen expression in primary human chondrocytes.** Primary human chondrocytes were cultured and stained with antibodies to Collagen VI (green), Collagen II (red) and Nuclear Marker Vibrant^TM^DyeCycle^TM^ Violet Stain. Shown are monolayer-expanded human primary cultured (**A**) day 1 no ascorbate (D1 No Asc), (**B**) day 3 ascorbate (D3 Asc), (**C**) day 7 no ascorbate (D7 No Asc) and (**D**) day 7 ascorbate (D7 Asc). (**E**) Alginate-encapsulated chondrocytes day 1 no ascorbate (D1 No Asc), (**F**) day 3 ascorbate (D3 Asc), (**G**) day 7 no ascorbate (D7 No Asc) and (**H**) day 7 ascorbate (D7 Asc). Sections of chondrocytes released from alginate and re-embedded in agarose (**I**) day 7 no ascorbate (D7 No Asc) and (**J**) day 7 ascorbate (D7 Asc). Scale bar represents 12.5 µm in panels A-D and 25 µm in E-J.


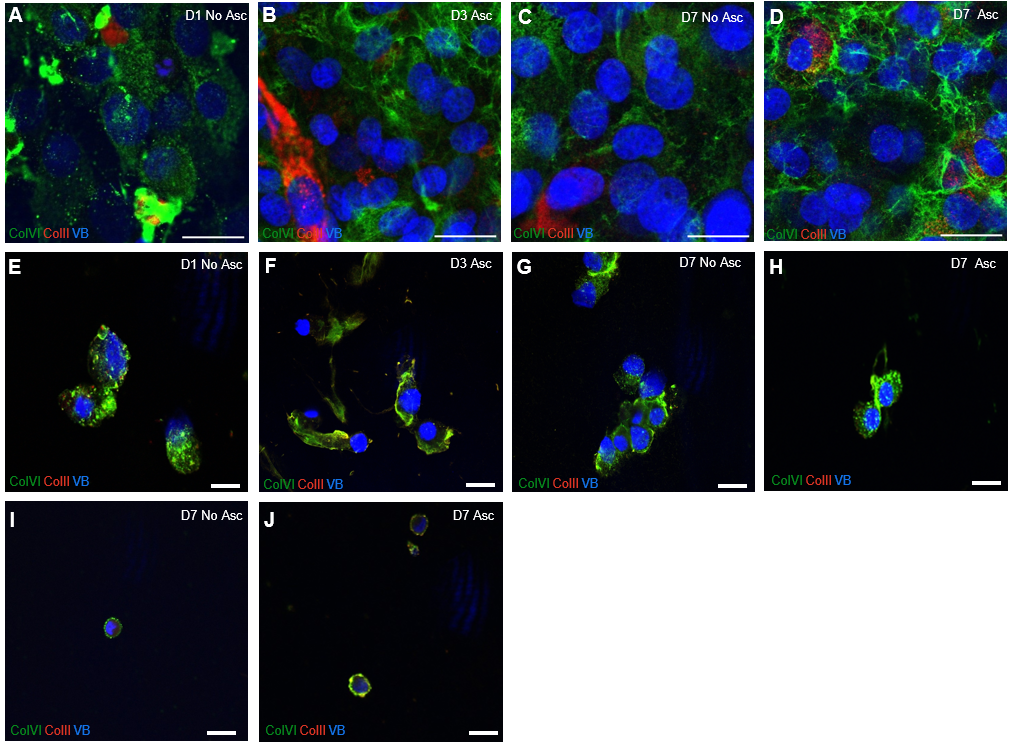


# Figure S4

**Expression of Collagens VI and II in bovine chondrocytes.** Bovine chondrocytes were cultured and stained with antibodies to Collagen VI (green), Collagen II (red) and Nuclear Marker Vibrant^TM^DyeCycle^TM^ Violet Stain. Shown are monolayer-expanded bovine chondrocytes cultured (**A**) day 1 no ascorbate (D1 No Asc), (**B**) day 3 ascorbate (D3 Asc), (**C**) day 7 no ascorbate (D7 No Asc) and (**D**) day 7 ascorbate (D7 Asc). (**E**) Alginate-encapsulated chondrocytes day 1 no ascorbate (D1 No Asc), (**F**) day 3 ascorbate (D3 Asc), (**G**) day 7 no ascorbate (D7 No Asc) and (**H**) day 7 ascorbate (D7 Asc). Sections of chondrocytes released from alginate and re-embedded in agarose (**I**) day 7 no ascorbate (D7 No Asc) and (**J**) day 7 ascorbate (D7 Asc). Scale bar represents 12.5 µm in panels A-D and 25 µm in E-J.

#
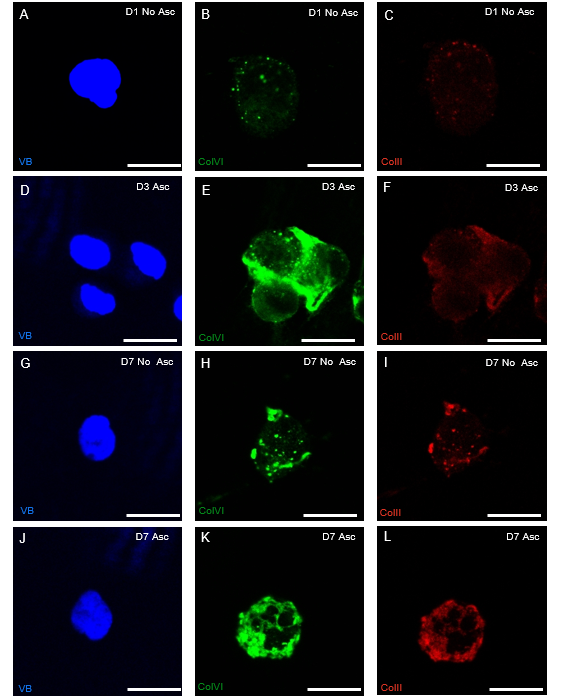


# Figure S5

**Expression of Collagens VI and II in primary chondrocytes.** Alginate encapsulated primary human chondrocytes were cultured and stained with antibodies to Collagen VI (green), Collagen II (red) and Nuclear Marker Vibrant^TM^DyeCycle^TM^ Violet Stain. Shown are single channel images. Panels **A–C** show Day 1 no ascorbate; panels **D–F** show Day 3 ascorbate; panels **G–I** show Day 7 no ascorbate; and panels **J–L** show Day 7 ascorbate. Scale bar represents 12.5 µm.

#
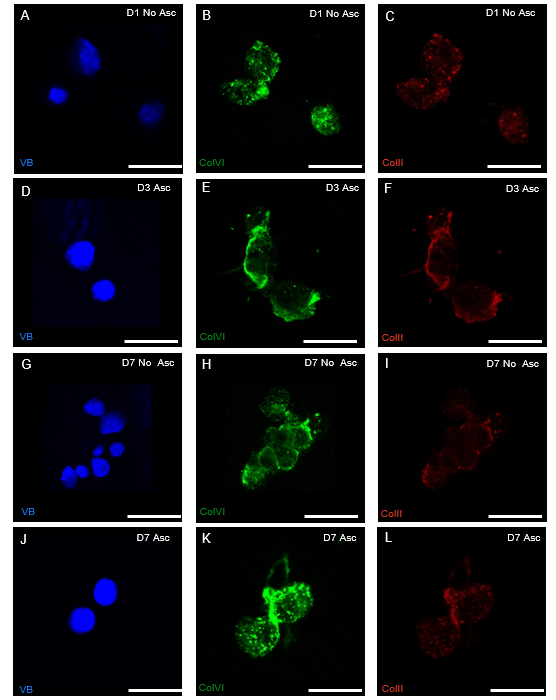


# Figure S6

**Expression of Collagens VI and II in bovine chondrocytes.** Alginate encapsulated bovine chondrocytes were cultured and stained with antibodies to Collagen VI (green), Collagen II (red) and Nuclear Marker Vibrant^TM^DyeCycle^TM^ Violet Stain. Shown are single channel images. Panels **A–C** show Day 1 no ascorbate; panels **D–F** show Day 3 ascorbate; panels **G–I** show Day 7 no ascorbate; and panels **J–L** show Day 7 ascorbate. Scale bar represents 12.5 µm.

#
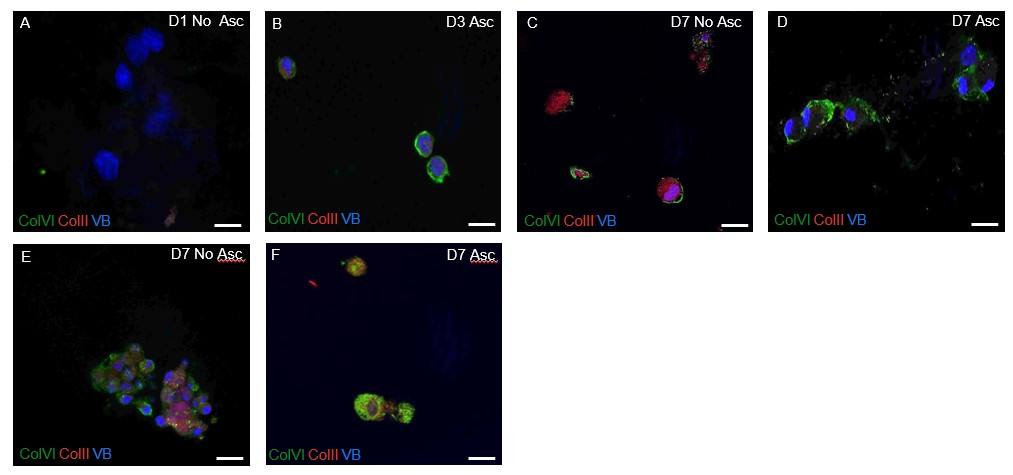
Figure S7

**Expression of Collagens VI and II in clusters of primary chondrocytes.** Primary chondrocytes were cultured and stained with antibodies to Collagen VI (green), Collagen II (red) and Nuclear Marker Vibrant^TM^DyeCycle^TM^ Violet Stain. Shown are clusters (**A**) Alginate-encapsulated chondrocytes day 1 no ascorbate (D1 No Asc), (**B**) day 3 ascorbate (D3 Asc), (**C**) day 7 no ascorbate (D7 No Asc) and (**D**) day 7 ascorbate (D7 Asc). Sections of chondrocytes released from alginate and re-embedded in agarose (**E**) day 7 no ascorbate (D7 No Asc) and (**F**) day 7 ascorbate (D7 Asc). Scale bar represents 25 µm.


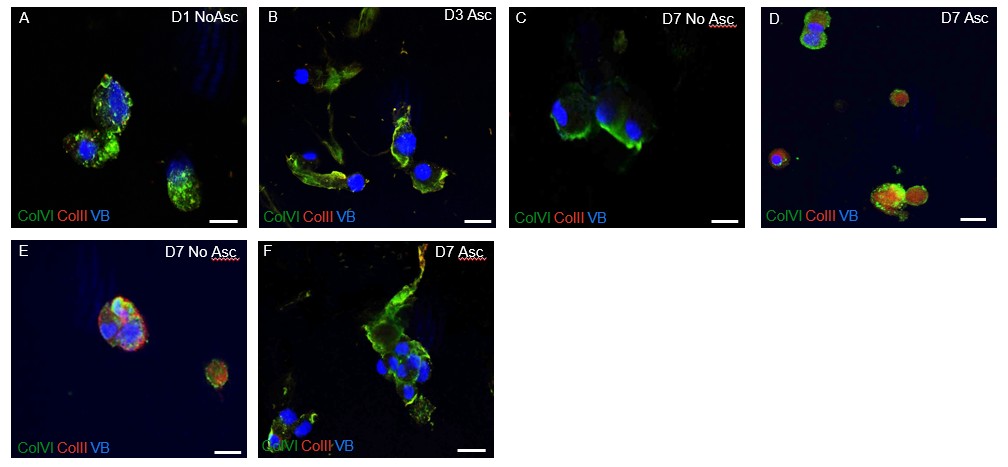


# Figure S8

**Expression of Collagens VI and II in clusters of bovine chondrocytes.** Bovine chondrocytes were cultured and stained with antibodies to Collagen VI (green), Collagen II (red) and Nuclear Marker Vibrant^TM^DyeCycle^TM^ Violet Stain. Shown are clusters (**A**) Alginate-encapsulated chondrocytes day 1 no ascorbate (D1 No Asc), (**B**) day 3 ascorbate (D3 Asc), (**C**) day 7 no ascorbate (D7 No Asc) and (**D**) day 7 ascorbate (D7 Asc). Sections of chondrocytes released from alginate and re-embedded in agarose (**E**) day 7 no ascorbate (D7 No Asc) and (**F**) day 7 ascorbate (D7 Asc). Scale bar represents 25 µm.


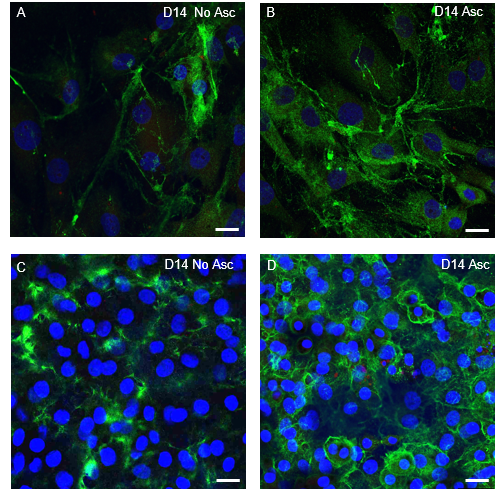


# Figure S9

**Expression of Collagens VI and II in primary and bovine chondrocytes monolayer on day 14.** Primary and bovine chondrocytes were cultured for 14 days with and without ascorbate and stained with antibodies to Collagen VI (green), Collagen II (red) and Nuclear Marker Vibrant^TM^DyeCycle^TM^ Violet Stain. **(A)** Primary human chondrocytes day 14 no ascorbate (D14 No Asc) **(B)** Primary human chondrocytes day 14 with ascorbate (D14 Asc) **(C)** Bovine chondrocytes day 14 no ascorbate (D14 NoAsc) **(D)** Bovine chondrocytes day 14 ascorbate (D14 Asc). Scale bar represents 25 µm.


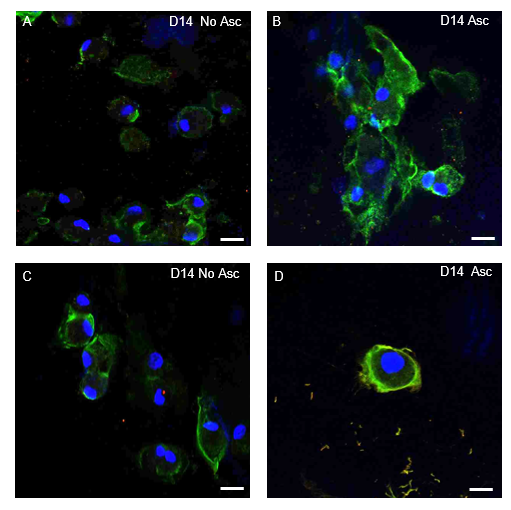


**Figure S10**

**Expression of Collagens VI and II in primary and bovine alginate encapsulated chondrocytes on day 14.** Alginate encapsulated primary and bovine chondrocytes were cultured for 14 days with and without ascorbate and stained with antibodies to Collagen VI (green), Collagen II (red) and Nuclear Marker Vibrant^TM^DyeCycle^TM^ Violet Stain. **(A)** Primary human chondrocytes day 14 no ascorbate (D14 No Asc) **(B)** Primary human chondrocytes day 14 with ascorbate (D14 Asc) **(C)** Bovine chondrocytes day 14 no ascorbate (D14 No Asc) **(D)** Bovine chondrocytes day 14 ascorbate (D14 Asc). Scale bar represents 25 µm.


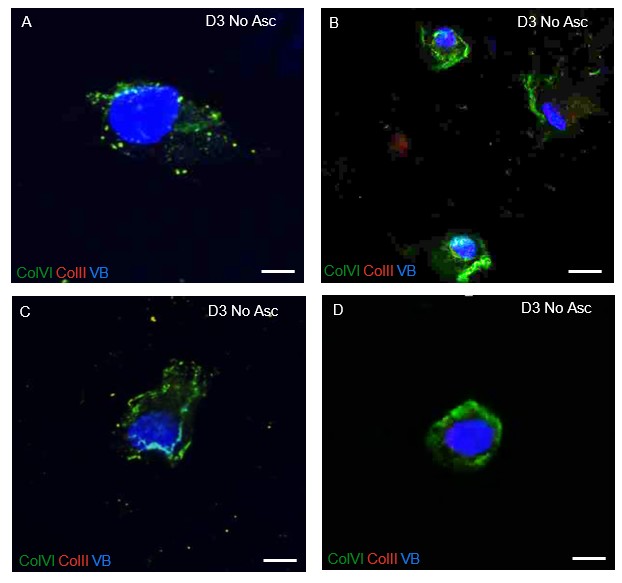


**Figure S11**

**Expression of Collagens VI and II in primary and bovine chondrocytes on day 3 without ascorbate.** Primary human and bovine chondrocytes were cultured and stained with antibodies to Collagen VI (green), Collagen II (red) and Nuclear Marker Vibrant^TM^DyeCycle^TM^ Violet Stain. Shown are **(A)** alginate encapsulated primary and **(B)** bovine chondrocytes on day 3 no ascorbate (D3 No Asc). **(C)** Primary and **(D)** bovine chondrocytes released from alginate beads, re-embedded in agarose on day 3 no ascorbate (D3 No Asc)

Scale bar represents 25 µm.


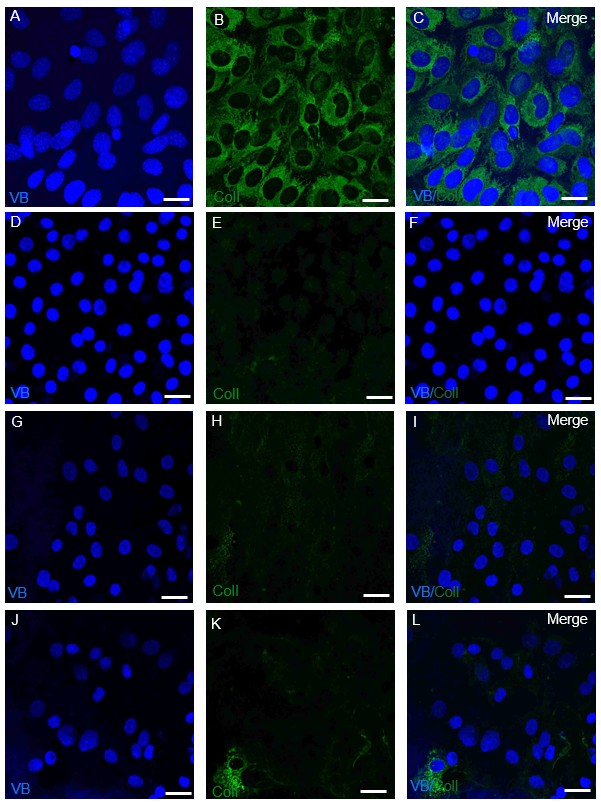


**Figure S12**

**Expression of Collagen I primary and bovine chondrocytes.** MC3T3 cells, primary human and bovine chondrocytes were cultured and stained with antibodies to Collagen I (green), and Nuclear Marker Vibrant^TM^DyeCycle^TM^ Violet Stain (blue). Shown are **(A)** monolayer MC3T3 cells stained for nuceli **(B)** Collagen I and **(C)** merged image. **(D)** Bovine chondrocytes stained for nuclei **(E)** Collagen I and **(F)** merged image. Primary human chondrocytes stained for nuclei **(G, J)** and showing variability with respect to Collagen I **(H, K)** and merged images **(I, L)**. Scale bar represents 25 µm.

**Supplemental Z-stack Movies**

Alginate encapsulated primary and bovine chondrocytes were cultured in complete media with 50μg/mL sodium L-ascorbate for 14 days in 5% CO_2_ at 37ºC. 100 μL of the alginate beads were cytospun onto single frosted adhesive slides (Tanner Scientific) using a Thermo Scientific Cytospin^TM^ 4Cytocentrifuge. Immunocytochemistry was then performed as described earlier. Cells were stained with antibodies to Collagen VI (green), Collagen II (red) and Nuclear Marker Vibrant^TM^DyeCycle^TM^ Violet Stain. Digital images were acquired on a Leica TCS SP8 confocal microscope and images were obtained with the Leica Application Suite Advanced Fluorescence software. To facilitate visualization of the PCM, 3D stack movies were created using ImageJ.

Supplemental Movie 1: Z-stack of Primary Human Chondrocytes encapsulated in Alginate

Supplemental Movie 2: Z-stack of Bovine Chondrocytes encapsulated in Alginate.
